# Umbilical Cord–Derived Mesenchymal Stem Cells Improve Ornidazole-Induced Asthenozoospermia in Rats via Activation of the AKT/mTOR Pathway

**DOI:** 10.1155/2024/3494652

**Published:** 2024-11-12

**Authors:** GaoBo Huang, Li Quan, Qi Li, Xiao Zhou, Mei Han, Fang Peng, YanFei Gong

**Affiliations:** ^1^Reproductive Center, Yueyang Central Hospital, Yueyang 414000, Hunan, China; ^2^Reproductive Center, Yueyang Maternal and Child Health Hospital, Yueyang 414000, Hunan, China; ^3^College of Life Sciences, Hunan Normal University, Changsha 410000, Hunan, China

**Keywords:** AKT/mTOR pathway, asthenozoospermia, autophagy, sperm quality, testicular injury, umbilical cord–derived mesenchymal stem cells

## Abstract

**Objective:** Mesenchymal stem cells (MSCs) have been highly confirmed for their critical role in the treatment of different diseases. This study focuses on the mechanism of umbilical cord–derived MSCs (UC-MSCs) in the treatment of ornidazole (ORN)-induced asthenozoospermia (AS) in rats via the AKT/mTOR pathway.

**Methods:** An animal model of AS was established in ORN-induced rats, followed by treatment of UC-MSCs and rapamycin (autophagy activator) or MK-2206 (AKT inhibitor). The sperm motility, concentration, and viability of rats were measured by an automatic sperm analyzer. Hematoxylin and eosin (HE) staining was conducted to observe the pathological injury of testicular tissue in rats. Terminal deoxynucleotidyl transferase dUTP nick end labeling (TUNEL) assay was utilized to evaluate the apoptosis rate of testicular cells. Western blot analysis was performed to determine the expression of apoptosis-related proteins, autophagy-related proteins, and AKT, p-AKT, mTOR, and p-mTOR. The rate of light chain 3 (LC3)–positive cells in testicular tissue was detected by immunohistochemistry (IHC).

**Results:** In ORN-induced AS rats, sperm motility, concentration, and viability as well as the number of mesenchymal cells and spermatogenic cells were significantly decreased, spermatogenic tubule space, apoptosis rate, and cleaved caspase-3, LC3II/I, Beclin-1, and LC3-positive cell rates were increased, and Bcl2 was downregulated. UC-MSCs could improve sperm quality and testicular injury in AS rats by inhibiting excessive autophagy. Besides, UC-MSCs could activate the AKT/mTOR pathway. Moreover, inhibition of the AKT/mTOR pathway partially reversed the therapeutic effect of UC-MSCs on ORN-induced AS rats.

**Conclusion:** UC-MSCs inhibit autophagy and improve sperm quality in AS rats through the AKT/mTOR pathway, highlighting a new idea for the treatment of AS.

## 1. Introduction

Infertility is an ongoing reproductive issue on a globe scale, and approximately half of the cases can be attributed to a male factor [1–3]. Asthenozoospermia (AS), featured with poor or absent motility of spermatozoa, holds accountable for male infertility to a large degree [4]. Dysfunctional sperm mitochondria and oxidative stress have been reported as possible causes of isolated AS [5]. In the context of oxidative stress, sperm autophagy has been observed to be activated while inhibited autophagy compromises sperm quality in contribution to cell apoptosis [6]. Microtubule-associated protein 1A/1B-light chain 3 (LC3) stands out as a specific marker for autophagosomes [7]. Notably, during autophagosome formation, a cytosolic form of LC3 (LC3-I) is enzymatically cleaved off a peptide segment, covalently binds to phosphatidylethanolamine, and is transformed into the autophagosome membrane type (LC3-II), and thus, the LC3-II/LC3-I ratio possesses the capacity to determine the level of autophagy [8]. Besides, the quantification of LC3-positive cells has been regarded as the gold standard for assessing the number of autophagosomes in cells [9]. Increasing research attention has been drawn to the implication of autophagy in male reproduction that abnormal autophagy triggers the defect of acrosome biogenesis and spermatid differentiation in the process of spermatogenesis [10–12]. Hereby, targeting autophagy modulation may provide novel insights into AS management.

Stem cell–based therapeutic strategies have emerged as regenerative medicine against reproductive disorders considering its self-renewal capacity and differentiation ability [13]. For instance, adipose-derived mesenchymal stem cells (MSCs) have been examined to preserve the viability and quality of post-thaw dog sperm [14]. Of note, umbilical cord–derived MSCs (UC-MSCs), characterized by painless extraction procedure and noncontroversial sources, have been demonstrated as a promising approach to female infertility triggered by premature ovarian failure [15, 16]. Also, multiple functional studies have confirmed the potential therapeutic application of UC-MSCs to restoring fertility in female population due to ovarian dysfunction or endometrial injury [17–19]. In addition, injection of MSCs has been indicated to improve sperm functions impaired by testicular torsion–detorsion through regulation on the balance between glycogenesis and glycolysis [20]. More importantly, available evidence has attached the importance of the phosphoinositide 3-kinase/protein kinase B/mammalian target of rapamycin (PI3K/AKT/mTOR) signaling pathway to the motility and quality of sperm [21]. The proliferation and differentiation of spermatogonium, spermatocyte, and spermatogenic cells have been documented to be mediated by mTOR [22]. Evidence has accumulated for the pivotal part of the AKT/mTOR signaling pathway playing in cell apoptosis regulated by UC-MSCs as well [23, 24]. Given that the modulatory effect of UC-MSCs on AS by affecting autophagy through the AKT/mTOR signaling pathway remains essentially unknown, the study aims to decipher the underlying mechanism by which UC-MSCs exerts inhibitory effect on autophagy to improve the sperm quality, providing theoretical basis for clarifying AS pathogenesis as well as novel insight into AS management.

## 2. Materials and Methods

### 2.1. Ethics Statement

All involved animal experiments were ratified by Yueyang Central Hospital. We made considerable efforts to minimize the animal quantity and their pain.

### 2.2. Funding

This work was partially supported by grants from Project Fund of Yueyang Central Hospital (YYSZXYN2022014) and Basic Research Project of Yueyang (2069999).

### 2.3. Cell Culture

UC-MSCs (ATCC, PCS-500-010) were cultured in human MSC serum-free medium (CM-SC01, Pricella, Wuhan, Hubei, China). In brief, serum-free MSC growth supplement (CM-SC01, Pricella) was added into the basal medium at 1:100 to formulate human MSC complete medium. When the cell density reached 70%–80%, the cells were trypsinized and subcultured. The cells were identified without bacterial, fungal, and mycoplasma contamination. Cells at passage 3 to 5 were used in the following experiments.

### 2.4. Experimental Animals

Sexually mature male Sprague Dawley (SD) rats (weight: 330–370 g, purchased from the SJA Laboratory Animal Co., Ltd., Changsha, Hunan, China) were raised at 24 C± 2°C and housed by groups in separate cages under 12 h light:12 h darkness cycle with access to food and water ad libitum.

### 2.5. Animal Model Establishment

The rat model of AS was constructed by intragastric administration of ORN [25]. In the ORN group, ORN dispersible tablets (H20040375, China Meheco Topfond Pharma Co., Ltd., Henan, China) were first made into powder by grinding machine and dissolved in 1% (w/v) sodium carboxymethylcellulose (CMC-Na) (Millipore, Sigma) in water. Then, rats were fed with ORN (400 mg per kg of body weight) once a day by oral gavage for 14 days. Control rats received 1% (w/v) CMC-Na in water without ORN throughout the experiment.

### 2.6. Animal Treatment

SD rats were assigned into the following six groups (*n* = 6/each group): Con group; ORN group—AS rat model was established by intragastric administration of ORN; ORN + UC-MSCs group—both testicles of AS rats were injected locally with UC-MSCs (3 × 10^4^ cells/side; resuspended in 20 *μ*L phosphate-buffered saline (PBS)) [20, 26]; ORN + levocarnitine (LEV) group—AS rats were orally administered with 100 mg/kg LEV once a day for 4 weeks [27]; ORN + UC-MSCs + rapamycin group—AS rats were locally injected with UC-MSCs in testicle and intraperitoneally injected with 5 mg/kg rapamycin once a day for 4 weeks [28]; and ORN + UC-MSCs + MK-2206 group—AS rats were locally injected with UC-MSCs in testicle and fed with 100 mg/kg MK-2206 by gavage (3 times a week for 4 weeks) [29]. All dosing was done after modeling of AS. Among them, each testicle of rats in the ORN group was locally injected with 20 *μ*L PBS [20, 26]; LEV oral liquid (national medicine approved number: H19990372) is a conventional drug for the treatment of AS, purchased from Northeast Pharmaceutical Group Shenyang First Pharmaceutical Co., Ltd. (Shenyang, China); rapamycin (HY-10219) is an autophagy activator and MK-2206 (HY-108232) is a selective AKT phosphorylation inhibitor, both purchased from MedChemExpress (Shanghai, China).

Twenty-four hours after the last administration, testicle and epididymis samples of euthanized rats were collected after intraperitoneal excessive injection of 5% pentobarbital sodium (100 mg/kg). Tissue sections of the left testicle were made for HE staining, TUNEL assay, and IHC, and tissue homogenates of the right testicle were made for western blot detection, and sperm quality was detected on the bilateral epididymis.

### 2.7. Sperm Quality Measurement

The bilateral epididymis was incubated at 37°C for 30 min in Ham's F10 culture medium which was preheated in the water bath box until the epididymis sperm was fully dissociated. An automatic sperm analyzer (WLJY-9000, Qisheng (Shanghai) Medical Instrument Co., Ltd., Shanghai, China) was used to detect sperm motility, concentration, and viability [30].

### 2.8. HE Staining

Rat testicle specimens were fixed in 4% paraformaldehyde (P0099, Beyotime, Shanghai, China) for 48 h, dehydrated with ethanol, immersed with conventional wax, cut into 4 *μ*m thick continuous sections, and stored at room temperature. Histological analysis of testicular was performed using the HE staining kit (Solarbio, Beijing, China) [30].

### 2.9. TUNEL Assay

TUNEL apoptosis detection kit (C1098, Beyotime) was used to detect the apoptosis rate of testicular cells in each group. A microscope (Olympus, Japan) was used for observation and photographing.

### 2.10. IHC

The expression of LC3 in rat testicular tissues was detected by IHC. In short, the tissue specimens were fixed, paraffin-embedded, and cut into 4 *μ*m sections. The sections were dewaxed, rehydrated, sealed with hydrogen peroxide, heated with EDTA solution, and subjected to antigen repair. The primary antibody LC3 (1:200, ab48394, Abcam, Cambridge, UK) was added and incubated at 4°C overnight. Then it was incubated with secondary antibody goat anti-rabbit immunoglobulin G (IgG) H&L (HRP) (1:1000, ab6721, Abcam) at 37°C for 120 min [31]. Statistical analysis was performed using ImageJ (National Institutes of Health, Bethesda, Maryland, USA) to calculate the proportion of LC3-positive cells.

### 2.11. Western Blot Analysis

The total protein was extracted using RIPA lyse buffer (Beyotime) containing protease inhibitors (Roche, Complete Mini, Basel, Switzerland) and quantified using BCA assay kit (Beyotime). Then, 50 *μ*g protein was loaded onto a 10% SDS-PAGE and transferred to a PVDF membrane (Millipore, Billerica, MA, USA). At room temperature, the membrane was blocked with Tris-buffered saline containing 5% skim milk Tween-20 (TBST, Beyotime) and incubated with the primary antibodies at 4°C overnight. After washing, the membrane was incubated with the secondary antibody for 1 h. *β*-Actin was used as the internal parameter. Chemiluminescence kit (ECL Plus, Life Technology) was used to detect protein bands, and ImageJ (National Institutes of Health) was used for grayscale analysis. The relevant information of antibodies used in this study is shown in Table 1.

### 2.12. Statistical Analysis

All data were statistically analyzed and plotted using GraphPad Prism 8.01 (GraphPad Software Inc.) software. Measurement data were presented in the form of mean ± standard deviation. Independent sample *t*-test was used for comparison between two groups, and one-way ANOVA was used for comparison among multiple groups, followed by Tukey's multiple comparisons test. *p* was two-sided test, and *p* < 0.05 indicated a statistically significant difference.

## 3. Results

### 3.1. UC-MSCs Improve the Sperm Quality of ORN-Induced AS Rats

**Figure 1:**
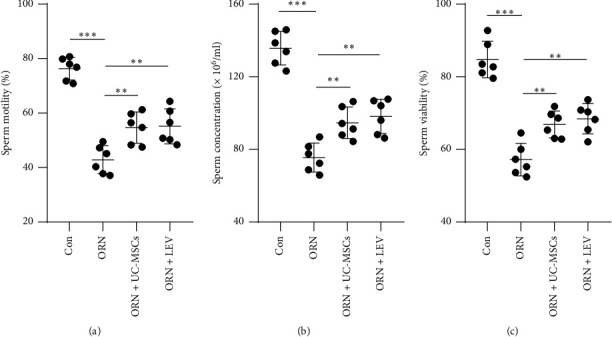
UC-MSCs improve the sperm quality of ORN-induced AS rats. (a) Detection of sperm motility. (b) Detection of sperm concentration. (c) Detection of sperm viability. *N* = 6. Measurement data were presented in the form of mean ± standard deviation. One-way ANOVA was used for comparison among multiple groups, followed by Tukey's multiple comparisons test. ⁣^∗∗^ *p* < 0.01; ⁣^∗∗∗^ *p* < 0.001.

To initially explore the role of UC-MSCs in AS, rats were induced by ORN to establish an animal model of AS, and then treated with UC-MSCs or LEV. The sperm motility, concentration, and viability of rats in each group were measured by an automatic sperm analyzer. Compared with the Con group, sperm motility, concentration, and viability in the ORN group were prominently decreased (Figures 1(a), 1(b), and 1(c), all *p* < 0.001). In comparison with the ORN-induced rats, the sperm motility, concentration, and viability of rats were notably elevated after UC-MSCs or LEV treatment (Figures 1(a), 1(b), and 1(c), all *p* < 0.01). Meanwhile, rats treated with UC-MSCs and LEV had no significant difference in sperm motility, concentration, or viability (Figures 1(a), 1(b), and 1(c), all *p* > 0.05). These results indicated that UC-MSCs improved the sperm quality of ORN-induced AS rats.

### 3.2. UC-MSCs Improve ORN-Induced Testicular Injury in AS Rats

**Figure 2:**
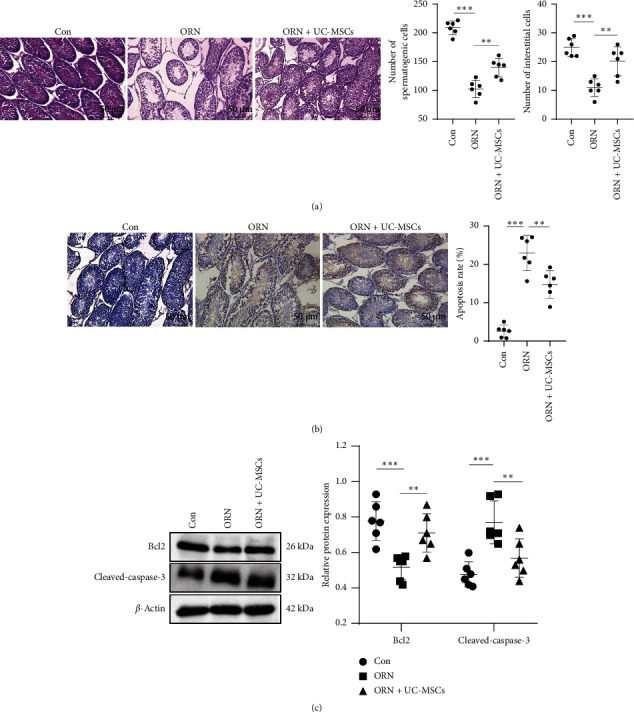
UC-MSCs improve ORN-induced testicular injury in AS rats. (a) The histopathological changes of testicular tissues were observed by HE staining. (b) TUNEL assay was used to detect the apoptosis rate of testicular cells. (c) Levels of apoptosis-related proteins Bcl2 and cleaved-caspase-3 were examined by western blot analysis. *N* = 6. Measurement data were presented in the form of mean ± standard deviation. One-way ANOVA was used for comparison among multiple groups, followed by Tukey's multiple comparisons test. ⁣^∗∗^ *p* < 0.01; ⁣^∗∗∗^ *p* < 0.001.

HE staining for observing the testicular injury of rats showed that no obvious pathological changes were observed in the testicular tissue of rats in the Con group, and the cells were arranged in an orderly and compact manner with normal morphology and structure. Compared with the Con group, the spermatogenic tubule space in the testicle tissue of rats increased, and the number of stromal cells and spermatogenic cells decreased in the ORN group (Figure 2(a), all *p* < 0.001). The above symptoms were significantly improved after UC-MSCs treatment in comparison to the ORN group (Figure 2(a), all *p* < 0.01). As displayed in Figures 2(b) and 2(c), we found that the apoptosis rate and cleaved caspase-3 protein expression were increased, while Bcl2 protein expression was decreased in the testicular tissue of rats in the ORN group versus the Con group (all *p* < 0.001). Compared with the ORN group, the apoptosis rate and cleaved caspase-3 protein expression in the testicular tissue of rats in the ORN + UC-MSCs group were significantly decreased, and the Bcl2 protein expression was markedly increased (all *p* < 0.001). These results confirmed that UC-MSCs improved ORN-induced testicular injury in AS rats.

### 3.3. UC-MSCs Ameliorate ORN-Induced Testicular Injury and Improve Sperm Quality in AS Rats by Inhibiting Excessive Autophagy

**Figure 3:**
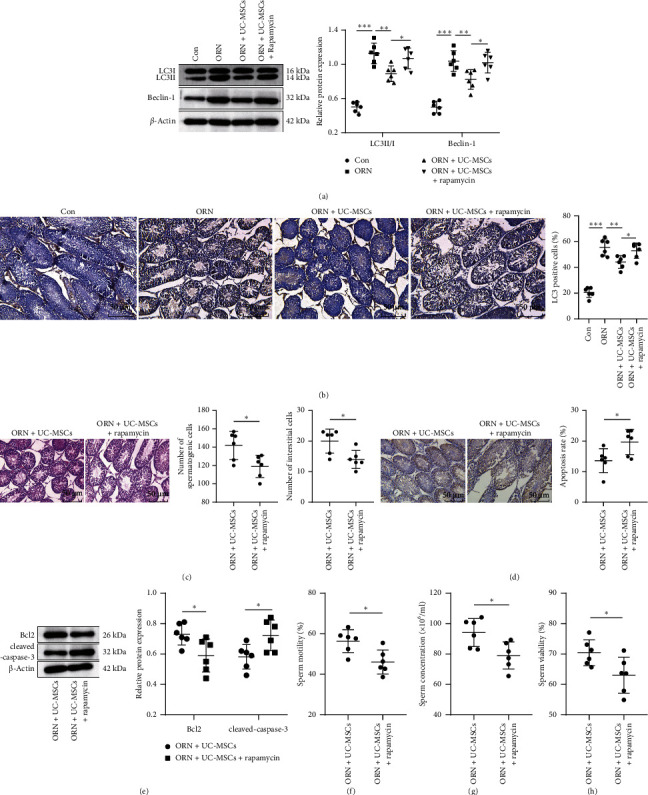
UC-MSCs mitigate ORN-induced testicular injury and improve sperm quality in AS rats by inhibiting excessive autophagy. (a) Western blot analysis was adopted to detect the LC3II/I and Beclin-1. (b) IHC was employed to examine the LC3-positive cell rate in the testicular tissue. (c) HE staining was implemented to observe the pathological changes of testicular tissue. (d) TUNEL assay was performed to evaluate the apoptosis rate of testicular cells. (e) Western blot analysis was used to test the levels of Bcl2 and cleaved caspase-3. (f) Detection of sperm activity. (g) Detection of sperm concentration. (h) Detection of sperm viability. *N* = 6. Data were expressed as mean ± standard deviation. One-way ANOVA was used in (a) and (b), and Tukey's multiple comparisons test was used for post hoc analysis. Independent sample *t*-test was used in (c)–(h). ⁣^∗^*p* < 0.05; ⁣^∗∗^ *p* < 0.01; ⁣^∗∗∗^ *p* < 0.001.

Accumulating evidence has confirmed that abnormal autophagy may lead to the defect of acrosome biogenesis and spermatid differentiation during spermatogenesis [10, 11]. Therefore, we tried to further explore whether UC-MSCs can improve ORN-induced testicular injury and sperm quality in AS rat by inhibiting excessive autophagy. AS rats were treated with UC-MSCs and intraperitoneally injected with 5 mg/kg rapamycin (autophagy activator). Western blot analysis and IHC revealed that compared with the Con group, expressions of LC3II/I and Beclin-1 and LC3-positive cells in testicular tissue of rats in the ORN group were significantly upregulated (Figures 3(a) and 3(b), *p* < 0.001); the expressions of LC3II/I and Beclin-1 and LC3-positive cells in the testicular tissue of AS rats after UC-MSCs treatment were significantly reduced in contrast to the ORN group (Figures 3(a) and 3(b), *p* < 0.01). After the use of autophagy activator, the expressions of LC3II/I and Beclin-1 and LC3-positive cells in rat testicular tissue were notably upregulated (Figures 3(a) and 3(b), *p* < 0.05). Further determination of testicular tissue injury, apoptosis rate, apoptosis-related protein expression, and sperm quality confirmed that compared with the ORN + UC-MSCs group, the number of rat stromal cells and spermatogenic cells in the ORN + UC-MSCs + rapamycin group was significantly reduced (Figure 3(c), all *p* < 0.05), apoptosis rate and cleaved caspase-3 expression were markedly upregulated (Figures 3(d) and 3(e), *p* < 0.05), Bcl2 expression was distinctly downregulated (Figure 3(e), *p* < 0.05), and sperm motility, concentration, and viability were decreased significantly (Figures 3(f), 3(g), and 3(h), *p* < 0.05). Altogether, UC-MSCs improved ORN-induced testicular injury and sperm quality in AS rats by suppressing excessive autophagy.

### 3.4. UC-MSCs Activate the AKT/mTOR Pathway

**Figure 4:**
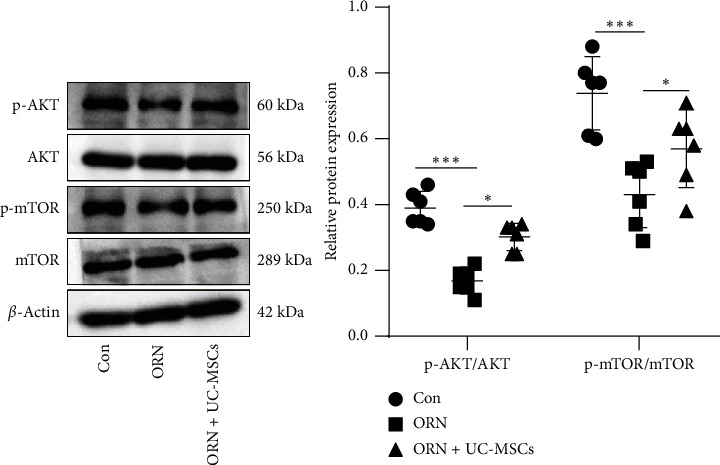
UC-MSCs activate the AKT/mTOR pathway. The protein levels of AKT, p-AKT, mTOR, and p-mTOR in testicular tissue were detected by western blot analysis. *N* = 6. Measurement data were presented in the form of mean ± standard deviation. One-way ANOVA was used for comparison among multiple groups, followed by Tukey's multiple comparisons test. ⁣^∗∗^ *p* < 0.01; ⁣^∗∗∗^ *p* < 0.001.

Accumulating studies have shown that the AKT/mTOR signaling pathway, a classic autophagy-related pathway, is related to sperm motility and sperm quality [21, 22, 32, 33]. To further explore whether UC-MSCs inhibited excessive autophagy of testicular cells by activating the AKT/mTOR pathway, the expression of AKT, p-AKT, mTOR, and p-mTOR proteins in testicular tissue of rats was detected with the results showing that compared with the Con group, p-AKT/AKT and p-mTOR/mTOR levels in testicular tissue of rats in the ORN group were significantly downregulated (Figure 4, both *p* < 0.001); p-AKT/AKT and p-mTOR/mTOR levels in testicular tissue of rats in the ORN + UC-MSCs group were significantly upregulated in comparison to the ORN group (Figure 4, both *p* < 0.05). These results suggested that UC-MSCs activated the AKT/mTOR pathway.

### 3.5. Inhibition of the AKT/mTOR Pathway Partially Reverses the Therapeutic Effect of UC-MSCs on ORN-Induced AS Rats

**Figure 5:**
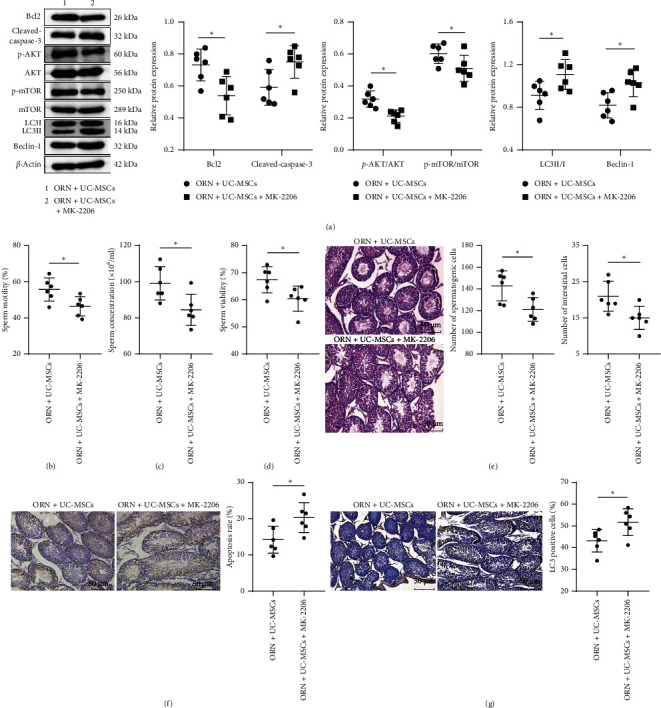
Inhibition of the AKT/mTOR pathway partially reverses the therapeutic effect of UC-MSCs on ORN-induced AS rats. (a) Levels of AKT, p-AKT, mTOR, p-mTOR, Bcl2, cleaved caspase-3, LC3II/I, and Beclin-1 in testicular tissue were measured by western blot analysis. (b) Detection of sperm activity. (c) Detection of sperm concentration. (d) Detection of sperm viability. (e) HE staining was used to observe the pathological changes of testicular tissue. (f) TUNEL assay was performed to assess the apoptosis rate of testicular cells. (g) IHC was implemented to detect the LC3-positive cell rate in the testicular tissue. *N* = 6. Measurement data were presented in the form of mean ± standard deviation. Independent sample *t*-test was used for two-group comparison. ⁣^∗^*p* < 0.05.

To further verify the role of the AKT/mTOR pathway in UC-MSC treatment of ORN-induced AS rats, AS rats were treated with UC-MSCs and administered with 100 mg/kg MK-2206 (AKT inhibitor). Western blot analysis detection revealed that compared with the ORN + UC-MSCs group, the protein levels of p-AKT/AKT and p-mTOR/mTOR in testicular tissue of rats in the ORN + UC-MSCs + MK-2206 group were significantly decreased (Figure 5(a), both *p* < 0.05), indicating that we successfully inhibited the AKT/mTOR pathway activation. Subsequently, sperm quality, testicular injury, apoptosis rate, apoptosis-related proteins (Bcl2 and cleaved caspase-3), autophagy-related proteins (LC3II/I and Beclin-1), and LC3-positive cell rates were further measured. Results suggested that compared with the ORN + UC-MSCs group, the sperm quality of rats was significantly decreased (Figures 5(b), 5(c), and 5(d), all *p* < 0.05), the number of mesenchymal cells and spermatogenic cells in testicular tissue was notably decreased (Figure 5(e), all *p* < 0.05), apoptosis rate and cleaved caspase-3 expression were distinctly upregulated (Figures 5(a) and 5(f), both *p* < 0.05), Bcl2 was significantly downregulated (Figure 5(a), *p* < 0.05), and LC3II/I, Beclin-1, and LC3-positive cell rates were markedly upregulated (Figures 5(a) and 5(g), all *p* < 0.05) in the ORN + UC-MSCs + MK-2206 group. These results uncovered that inhibition of the AKT/mTOR pathway partially reversed the therapeutic effect of UC-MSCs on ORN-induced AS rats.

## 4. Discussion

It is estimated that approximately 70 million individuals are struggling with infertility across the world, yet the understanding on the molecular, cellular, and genetic factors in contribution to AS has not been fully answered [34, 35]. The findings collected from the murine model presented in this study supported that UC-MSCs exerted inhibitory effect on cell autophagy via the AKT/mTOR signaling pathway, by which the sperm quality of AS rats was partially restored.

Based on the preliminary exploration, the sperm quality of rats administered with ORN by gavage was significantly improved after local injection of UC-MSCs into the testicle, accompanied by ameliorated testicular injury caused by ORN in AS rats. Largely in agreement with our observations and evaluations, a beneficial effect of adipose-derived MSCs conditioned medium has been observed on sperm parameters [36]. Moreover, testis ischemia-reperfusion injury has been elucidated to be alleviated by conditioned medium of bone marrow–derived MSCs along with reduced abnormal sperm rate and elevated viability [37]. Evidence has been presented demonstrating that semen quality and testosterone levels of male rats are significantly ameliorated by treatment of MSCs derived from the bone marrow [38]. It is noted that canine adipose-derived MSCs provide effective protection to the post-thaw dog sperm against cryoinjury in a dose-dependent manner [14]. According to a functional report, MSCs harbor the property of increasing sperm number and improving sperm motility within 3 days following testicular torsion/detorsion injury [20]. Thereupon, additional attempts are required to probe into the effect of UC-MSCs–based therapy against AS in the light of various concentrations and time duration.

Furthermore, the experimental data revealed that the sperm quality restored by UC-MSCs relied on excessive autophagy inhibition as evidenced by reduced cells positive for LC3II/I, Beclin-1, and LC3 in testicular tissues of AS rats. Autophagy, facilitating cell survival and guiding cell function through degradation of excess proteins and organelle, is of great importance during spermatogenesis that abnormal autophagy leads to male reproductive toxicity exacerbation and even infertility [39, 40]. Similar to our results, oxidative stress has been found to be stimulated and autophagic vacuole number is increased in mouse GC-1 spg cell lines induced with testicular cell injury where autophagy-related LC3-II, Beclin 1, Atg5, and LC3-II/LC3-I levels are elevated [41]. Also, a mechanistic study has highlighted that diminished sperm count and impaired sperm motility following exposure to methylmercury are concomitant with upregulation of LC3-II and Beclin-1 as well as inhibited PI3K/AKT/mTOR signaling pathway [42]. The subsequent mechanistic investigation in our study suggested that the AKT/mTOR signaling pathway was activated by UC-MSCs, the therapeutic action of which on ORN-evoked AS was partially reversed when the AKT/mTOR signaling pathway was inhibited. Coherently, the activated PI3K/AKT pathway has been reported concurrent with increased sperm mobility in patients with AS [43]. The PI3K/AKT/mTOR signaling pathway in mouse testis has been suggested to be inhibited by aflatoxin B1, which can be responsible for severely impaired testicular development and spermatogenesis [44]. There is convincing evidence of the interplay between the PI3K/AKT/mTOR signaling pathway and retinoic acid for spermatogonial differentiation [45]. Additionally, apoptosis and autophagy of testicular tissues have been indicated to be promoted by nano-copper, which is hazardous to the reproductive system of male rats, through inhibition on the AKT/mTOR signaling pathway [46]. As shown in this paper, the addition of MK-2206 for inhibition on the AKT/mTOR signaling pathway demonstrated partially countervailing therapeutic action of UC-MSCs on ORN-induced AS rats, further validating our findings.

To conclude, the present study establishes a preliminary understanding on the UC-MSCs–elicited improvement of sperm quality in AS rats by inhibiting autophagy through the AKT/mTOR signaling pathway. Nevertheless, this study only simply assessed sperm quality via sperm motility, sperm concentration, and sperm viability, with subsequent studies needed for further observation of sperm morphology by sperm smears to validate the improvement effect of UC-MSCs on sperm quality. Meanwhile, we did not consider whether the local stimulation of the testicles after local injection into the testicles would affect the section observation or protein, which would be further improved in subsequent studies. Further investigation is expected to study whether oxidative stress is involved in fertility restoration induced by UC-MSCs considering the multifaceted interactions between apoptosis and autophagy modulated by oxidative stress in spermatogenesis, thereby offering potential assisted reproductive technologies against AS in the future. More importantly, to extrapolate the findings obtained from murine models to clinical setting requires caution as a result of the evolutionary divergence regarding pluripotency of stem cell–based therapy for infertility between animals and humans.

## Figures and Tables

**Table 1:** Antibodies used in western blot analysis.

**Primary antibodies**	**Cat No.**	**Dilution**	**Company**

Bcl2	ab196495	1:1000	Abcam
Cleaved caspase-3	GTX86952	1:1000	GeneTex
LC3	ab192890	1:2000	Abcam
Beclin-1	ab207612	1:2000	Abcam
p-AKT	ab38449	1:1000	Abcam
AKT	ab8805	1:500	Abcam
p-mTOR	GTX132803	1:1000	Abcam
mTOR	ab32028	1:1000	Abcam
*β*-Actin	ab8227	1:1000	Abcam

**Secondary antibody**	**Cat No.**	**Dilution**	**Company**

Goat Anti-Rabbit IgG H&L (HRP)	ab6721	1:1000	Abcam

## Data Availability

The datasets supporting the conclusions of this article are included within the article.
